# An Exceptionally Rare Case of Acute Prostatitis Caused by Myroides odoratimimus Infection

**DOI:** 10.7759/cureus.88554

**Published:** 2025-07-22

**Authors:** Seiya Shiramizu, Hisami Aono, Kazunobu Aramaki, Emi Morinaka, Naohiro Fujimoto

**Affiliations:** 1 Urology, Local Incorporated Administrative Agency, Kurate Hospital, Kurate, JPN; 2 Urology, University of Occupational and Environmental Health, Kitakyusyu, JPN; 3 Clinical Laboratory, Local Incorporated Administrative Agency, Kurate Hospital, Kurate, JPN

**Keywords:** acute prostatitis, benign prostatic hyperplasia, multidrug-resistant environmental bacterium, myroides odoratimimus, wave

## Abstract

*Myroides odoratimimus* (*M. odoratimimus*) is a rare, multidrug-resistant bacterium that occasionally causes opportunistic infections in the environment. We report the first documented case of acute prostatitis caused by *M. odoratimimus* in a 95-year-old man following transurethral water vapor energy therapy for benign prostatic hyperplasia. The patient developed fever and prostatic tenderness postoperatively, with CT findings consistent with prostatitis. The initial empirical treatment with piperacillin/tazobactam was ineffective. Urine culture revealed a multidrug-resistant bacterium susceptible only to minocycline, which was identified as *M. odoratimimus* by matrix-assisted laser desorption/ionization time-of-flight mass spectrometry and 16S rRNA sequencing. Treatment with minocycline led to clinical improvement. This case underscores the importance of considering rare pathogens in patients with indwelling catheters or postoperative infections, particularly when standard antimicrobial therapies prove ineffective. Accurate microbiological diagnosis and appropriate treatment based on antimicrobial susceptibility testing are essential in managing such infections.

## Introduction

Acute prostatitis is an inflammatory condition of the prostate gland, commonly caused by Gram-negative bacteria, most frequently *Escherichia coli*. Indwelling urinary catheters, transurethral surgical procedures including water vapor energy therapy (WAVE), and instrumentation are recognized risk factors for the development of acute prostatitis. While typically responsive to antibiotic therapy, it can lead to severe complications if untreated or inadequately treated.

Infections caused by uncommon or rare organisms are of particular clinical interest, especially when they present diagnostic and therapeutic challenges. *Myroides* species are Gram-negative, non-fermenting bacilli commonly found in environmental sources such as water and soil [1]. Historically considered low-virulence opportunistic pathogens that primarily affect immunocompromised individuals, recent reports have demonstrated that *Myroides* species can also cause serious infections in immunocompetent hosts [2,3]. Among these, *Myroides odoratimimus* (*M. odoratimimus*) has been implicated in various infections, including urinary tract infections, soft tissue infections, and sepsis [2,4]. A major concern regarding *Myroides* infections is their intrinsic multidrug resistance, which limits therapeutic options [3,5].

We report a case of acute bacterial prostatitis caused by *M. odoratimimus* following WAVE for benign prostatic hyperplasia (BPH) in a patient with an indwelling bladder catheter. To our knowledge, this is the first documented case of prostatitis attributable to *M. odoratimimus*. The clinical course and microbiological findings are described in this section.

## Case presentation

A 95-year-old Japanese male with a history of hypertension and dementia underwent indwelling urinary catheter placement one month prior due to urinary retention associated with a markedly enlarged prostate (volume: 156 mL). He was referred to our institution for surgical management of BPH and scheduled to undergo transurethral WAVE therapy. Preoperative urinalysis revealed pyuria (WBC 50-99/high-power field) and bacteriuria; therefore, intravenous piperacillin/tazobactam (PIPC/TAZ) 4.5 g was administered twice daily for two days before surgery.

**Figure 1:**
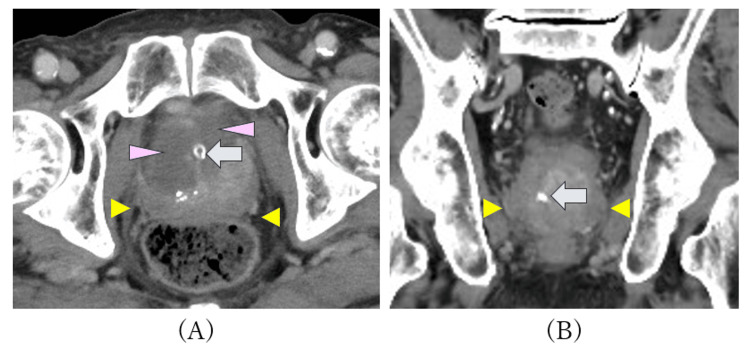
Axial (A) and coronal image (B) contrast-enhanced CT on postoperative day 6 Enhancement of the prostate and haziness of the periprostatic fat tissue (yellow arrowhead). Low-density area associated with the effect of WAVE procedure (purple arrowhead). Urethral catheter (blue arrow). CT: computed tomography, WAVE: water vapor energy therapy

On the first postoperative day, the patient developed a fever of 38.8°C. He reported chills and discomfort in the urethral and perineal areas. Physical examination revealed prostatic tenderness on digital rectal examination. There were no respiratory symptoms, abdominal pain, costovertebral angle tenderness, or signs of epididymitis. Laboratory findings (Table 1) showed leukocytosis, neutrophilia, and elevated CRP. Contrast-enhanced computed tomography (CT) revealed a low-density area within the prostate, likely attributable to the effects of WAVE therapy, along with enhancement of the prostatic parenchyma and periprostatic fat stranding, findings suggestive of prostatic inflammation (Figure 1). There was no evidence of infection in other organs.

**Table 1:** Blood laboratory test results on the day of fever onset White blood cells, neutrophils, and C-reactive protein levels were elevated compared to normal values. eGFR: estimated glomerular filtration rate, Na: sodium, K: potassium, Cl: chloride

Parameter	Result	Reference range
White blood cell count (/μL)	10,180	3,300-8,600
Neutrophils (%)	77.8	37.0-72.0
Lymphocytes (%)	10.3	18-59
Red blood cell count (×10^4^/μL)	362	435-555
Hemoglobin (g/dL)	11.2	13.7-16.8
Platelet count (×10^3^/μL)	196	158-348
C-reactive protein (mg/dL)	6.2	0-0.14
Total protein (g/dL)	6	6.6-8.1
Albumin (g/dL)	2.9	4.1-5.1
Aspartate aminotransferase (U/L)	17	13-30
Alanine aminotransferase (U/L)	9	10-42
Gamma glutamyl transferase (U/L)	54	13-64
Alkaline phosphatase (U/L)	77	38-113
Lactate dehydrogenase (U/L)	155	124-222
Blood urea nitrogen (mg/dL)	16.3	8-20
Creatinine (mg/dL)	1.25	0.65-1.07
eGFR (ml/min/1.73m^2^)	41.1	90-120
Na (mmol/L)	139	138-145
K (mmol/L)	4.3	3.6-4.8
Cl (mmol/L)	105	101-108

**Figure 2:**
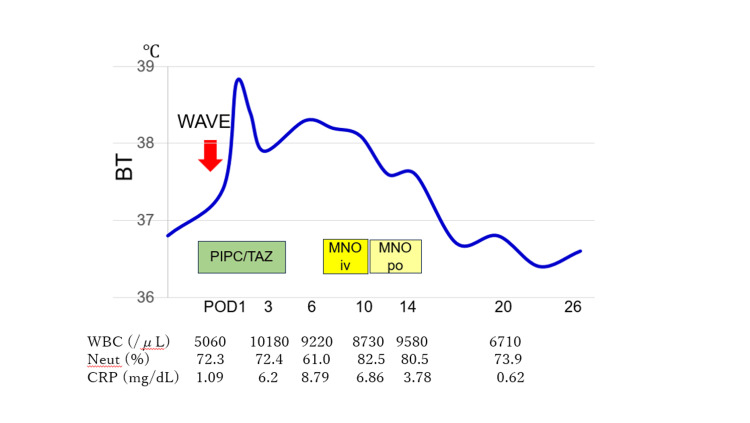
Patient’s clinical course BT: body temperature, CRP: C-reactive protein, MNO: minocycline, iv: intravenous administration, po: oral administration, Neut: percent of neutrophil count, PIPC/TAZ: piperacillin/tazobactam, POD: postoperative day, WBC: white blood cell count

Despite continued PIPC/TAZ administration for five days, the fever persisted. At this point during treatment, the urine culture obtained before surgery yielded 10^3 ^CFU/ml of *Chryseobacterium* sp. and *Corynebacterium* sp. using the WalkAway system (Beckman Coulter, Inc., Brea, CA, USA), while blood cultures were negative. Antimicrobial susceptibility testing (Table 2) demonstrated multidrug resistance, with susceptibility only to minocycline (MIC <2 μg/mL). Intravenous minocycline (100 mg twice daily for five days) led to resolution of fever and clinical improvement, followed by five additional days of oral minocycline. Inflammatory markers normalized (Figure 2), and the patient was discharged on postoperative day 28. Three months post-surgery, he regained spontaneous voiding, and the catheter was removed.

**Table 2:** Microbial susceptibility of isolated bacteria #1 and #2 were identified as *Chryseobacterium* sp. and *Corynebacterium* sp., respectively, using the WalkAway system. PCG: penicillin G, MPIPC: oxacillin, SBT/ABPC: sulbactam/ampicillin, AMPC: amoxicillin, PIPC: piperacillin, PIPC/TAZ: piperacillin/tazobactam, CEZ: cefazolin, CTM: cefotiam, SBT/CPZ: sulbactam/cefoperazone, CAZ: ceftazidime, CTRX: ceftriaxone, CFPM: cefepime, CZOP: cefozopran, CMZ: cefmetazole, CPDX-PR: cefpodoxime proxetil, CDTR-PI: cefditoren pivoxil, LMOX: latamoxef, IPM: imipenem, MEPM: meropenem, DRPM: doripenem, AMK: amikacin, ISP: isepamicin, ABK: arbekacin, CAM: clarithromycin, AZM: azithromycin, CLDM: clindamycin, MINO: minocycline, VCM: vancomycin, FOM: fosfomycin, LZD: linezolid, CPFX: ciprofloxacin, LVFX: levofloxacin, PZFX: pazufloxacin, STFX: sitafloxacin, MFLX: moxifloxacin, GRNX: garenoxacin, ST: sulfamethoxazole/trimethoprim, S: sensitive, I: intermediate, R: resistant

Antibiotics	#1	#2		Antibiotics	#1	#2
PCG	-	R		DRPM	R≥8	R
MPIPC	-	R		AMK	R≥64	S
SBT/ABPC	R	R		ISP	R	S
AMPC	R	R		ABK	-	-
PIPC	I=64	R		CAM	R	R
PIPC/TAZ	I=64	R		AZM	R	R
CEZ	R	R		CLDM	R	R
CTM	R	R		MINO	S≤2	S
SBT/CPZ	R≥64	R		VCM	-	S
CAZ	R≥32	R		FOM	I=16	R
CTRX	R≥64	R		LZD	-	-
CFPM	R≥32	R		CPFX	R≥4	R
CZOP	R≥32	R		LVFX	R≥8	R
CMZ	R	R		PZFX	R	R
CPDX-PR	R	R		STFX	R	S
CDTR-PI	R	R		MFLX	R	R
LMOX	R	R		GRNX	R	R
IPM	R	R		ST	R≥76	R
MEPM	R≥16	R				

**Figure 3:**
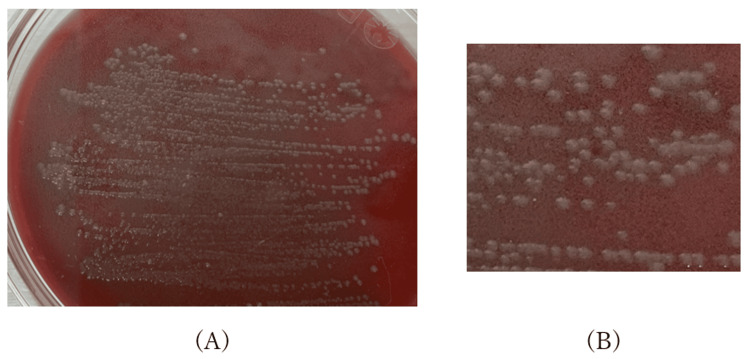
Bacterial culture on trypticase soy agar with 5% sheep blood (A) Whole view of the culture dish showing colony formation under aerobic conditions at 30°C. (B) Magnified view of the gray-colored colonies observed in the boxed area of (A).

To accurately identify the causative pathogen, preoperative urine specimens were cultured aerobically at 30°C on trypticase soy agar with 5% sheep blood (Becton Dickinson, Franklin Lakes, NJ, USA), producing gray-colored colonies (Figure 3). Matrix-assisted laser desorption/ionization time-of-flight mass spectrometry (MALDI-TOF MS) (MALDI Biotyper, MBT Compass Library Ver. 8; Bruker Daltonics, Japan) identified the isolate as *M. odoratimimus *with a confidence score >2.0. No other bacteria were detected. 16S rRNA gene sequencing (primers: 27F and 1494R) confirmed a 100% sequence identity with *M. odoratimimus*, thereby establishing the definitive diagnosis.

## Discussion

Although urinary tract infections caused by *M. odoratimimus* have been reported [4,6,7], no prior cases have explicitly documented genital tract infections, such as prostatitis or epididymitis. The present case represents the first report implicating *M. odoratimimus* in acute prostatitis.

Although bacterial isolation from prostatic tissue was not feasible, several lines of evidence strongly support the diagnosis of prostatitis caused by *M. odoratimimus*: (1) the urine culture performed before fever onset revealed only *M. odoratimimus* and the infection responded only to minocycline, which was the sole effective agent identified in susceptibility testing; (2) fever developed immediately after transurethral prostate surgery, likely facilitating bacterial entry into the prostate; (3) digital rectal examination revealed prostatic tenderness, a hallmark of acute bacterial prostatitis; and (4) CT findings were consistent with prostatitis, though postoperative changes could not be entirely excluded.

Furthermore, the possibility that *M. odontimimus* was detected in the urine represented colonization or contamination, which is unlikely, based on the following considerations. *M. odoratimimus* has been reported as a causative agent of urinary tract infections. To date, no cases of colonization of the urinary tract or prostate by *Myroides* species have been documented. In our case, *M. odoratimimus* was isolated as the sole organism at 10⁵ CFU/mL from an aseptically collected catheterized urine sample, and the clinical course matched the antimicrobial susceptibility profile. These observations provide strong evidence that *M. odontimimus* acted as a true pathogen rather than representing colonization or contamination.

While *M. odoratimimus* is generally considered a low-virulence organism, advanced age and the presence of an indwelling catheter likely contributed to this patient’s increased susceptibility to infection. Detection of *M. odoratimimus* in urine before urogenital surgery may portend a risk of postoperative complications. Preoperative eradication is therefore recommended, especially in immunocompromised patients or those with catheters. However, the bacterium’s multidrug resistance presents a significant challenge. In the case series reported by Ktari [6] and Kutlu et al. [7], *M. odoratimimus* demonstrated resistance to all tested antimicrobial agents in every case. In our case, as well as in four cases reported by Khan et al. [4], minocycline was the only agent demonstrating activity. These findings underscore the necessity of early culture and susceptibility testing in managing infections caused by rare, drug-resistant pathogens.

The WalkAway system misidentified the pathogen as *Chryseobacterium* sp. and *Corynebacterium* sp., likely due to overlapping biochemical profiles. *M. odoratimimus*, *Chryseobacterium *sp., and non-fermenting *Corynebacterium* sp. share several biochemical characteristics, including oxidase positivity, non-fermentation of glucose, and catalase positivity, complicating differentiation by automated systems. In addition, since the WalkAway system identifies organisms based on database matching, it may fail to accurately identify rare bacterial species. In suspected cases of rare infections, advanced techniques such as MALDI-TOF MS or 16S rRNA sequencing are essential for accurate pathogen identification.

## Conclusions

This case illustrates that *M. odoratimimus*, although rare, should be considered a potential causative organism of prostatitis, particularly in high-risk patients. Accurate microbiological diagnosis and susceptibility-guided antibiotic therapy are critical for successful management. Clinicians should remain vigilant when encountering unusual clinical presentations or lack of response to empiric therapy and initiate prompt microbiological evaluation to ensure appropriate treatment.
